# The prophylactic use of C1 esterase inhibitor in HAE patients undergoing invasive procedures

**DOI:** 10.1186/1710-1492-10-S2-A15

**Published:** 2014-12-18

**Authors:** Rachel Harrison, Stephanie Santucci, Geneviève Gavigan, Jacob Karsh, William H Yang

**Affiliations:** 1 Allergy and Asthma Research Centre, Ottawa, ON, Canada; 2 University of Ottawa Medical School, Ottawa, ON, Canada

## Background

For a patient with Hereditary Angioedema (HAE), physiological and/or psychological stress can cause insufficient control of local inflammatory pathways. This leads to complement and contact system activation and excess bradykinin resulting in angioedema. Therefore, an invasive procedure or surgery can trigger an HAE attack; this in turn can cause further medical complications and pose an added danger to the post-procedure patient. C1 inhibitor, Berinert®, was approved in the US and Canada in 2009 and 2010, respectively, for the treatment of acute attacks. In April 2013, Berinert® was approved in Europe for short-term prophylaxis prior to medical, dental, or surgical procedures to prevent HAE attacks. Currently, Berinert® is not approved in Canada or the US for prophylaxis. We aim to demonstrate the effectiveness of C1 esterase inhibitor, Berinert®, as a prophylactic treatment for HAE patients undergoing invasive procedures.

## Method

A retrospective chart review from our Canadian Tertiary Care Allergy and Asthma Clinic of our entire database of HAE patients was performed.

## Results

Between 1997 and June 2014, C1 esterase inhibitor for prophylactic use was administered prior to invasive procedures. There were a total of 28 procedures, performed on 15 patients.

The 28 procedures breakdown as follows: 9 dental surgeries, 3 open heart surgeries, 8 other surgical procedures, 3 child birth, 5 invasive procedures

In all 28 procedures, there was no incidence of post-procedure HAE attacks after prophylactic administration of C1 esterase inhibitor.

## Conclusion

We found that C1 esterase inhibitor decreased the incidence of post-procedure HAE attacks and was an effective prophylactic treatment.

